# Dr. Hammond’s Letter

**Published:** 1893-11

**Authors:** 


"THE FUNCTION OF THE PHARMACIST," an editorial in our August number, Dr. Hammond claims, does him an injustice. He rightly assumes the TEXAS MEDICAL JOURNAL would not intentionally do him a wrong; and in his letter published elsewhere in this issue, he points out wherein we were mistaken in regard to his position and relation to the animal extracts.

We were under the impression he had a proprietary interest in the formula for making the extracts, and sought to monopolize the sale, after having ostensibly given the formula to the profession; hence our denunciation of those journals which, from our understanding of the matter, appeared to be endorsing and sustaining him in what was, looked at from our point of view, clearly an unethical proceeding,—in fact—quackery. Our contention was that no physician could, consistently with the code, be the proprietor of a protected formula, and engage in the sale of such protected products; that it was the legitimate function of the manufacturing pharmacists to manufacture such products, when the formula had been given to the profession, and we saw no reason why he should object to Parke, Davis & Co., or any other firm manufacturing the extracts, except the selfish one just stated. Dr. Hammond assures us he has not, and has never had a proprietary interest in his formula, and we give place to his letter with pleasure, as fully explaining his position. We disclaim any wish or intention to do him, or the other party to the controversy, any injustice; we simply stated the case as we understood it at the time.

